# High‐Pressure Synthesis of Quantum‐Stabilised Manganese Superhydride MnH_7_

**DOI:** 10.1002/anie.9526719

**Published:** 2026-08-22

**Authors:** Mikhail A. Kuzovnikov, Hannah A. Shuttleworth, Israel Osmond, Huixin Hu, Elvita Meskauskaite, Bole Chen, Callum Stevens, Andrew Huxley, Andreas Hermann, Miriam Peña‐Alvarez, Ross T. Howie

**Affiliations:** ^1^ Centre for Science at Extreme Conditions the University of Edinburgh Edinburgh UK; ^2^ Center for High Pressure Science and Technology Advanced Research Shanghai China; ^3^ School of Electronic Science and Engineering Chongqing University of Posts and Telecommunications Chongqing China

**Keywords:** ab initio calculations, high‐pressure chemistry, high‐temperature chemistry, hydrides, manganese, X‐ray diffraction

## Abstract

Through high‐temperature, high‐pressure diamond anvil cell studies combined with in situ synchrotron powder x‐ray diffraction, we investigate the reactivity between manganese and hydrogen up to 182 GPa. Laser heating manganese in an $H_{2}$ medium at 118 GPa yields body‐centred tetragonal ${\mathrm{MnH}}_{7}$, which has the highest hydrogen content ever observed in the binary hydrides of group IV–XII transition metals. In addition, we also observe two other new hydrides as minor phases, ${\mathrm{MnH}}_{∼2}$ with a tetragonal crystal structure and cubic ${\mathrm{MnH}}_{3.67}$, formed at the pressures of 105 and 118 GPa, respectively. Upon decompression, ${\mathrm{MnH}}_{3.67}$ and ${\mathrm{MnH}}_{7}$ remain stable down to ∼30 GPa before decomposing into the monohydride. Our ab initio quantum chemistry computations employing density‐functional theory reveal that the key stabilisation factor of ${\mathrm{MnH}}_{3.67}$ and ${\mathrm{MnH}}_{7}$ is zero‐point energy, however, no isotope effect is observed on the crystal structures of the deuterated samples.

## Graphical abstract

Superhydrides are a rarity in transition metal hosts. Through high‐pressure, high‐temperature diamond anvil cell studies, the most hydrogen‐rich transition metal hydride, ${\mathrm{MnH}}_{7}$ has been synthesised. Density functional theory calculations demonstrate that zero‐point energy stabilises the crystalline structure that ${\mathrm{MnH}}_{7}$ adopts.

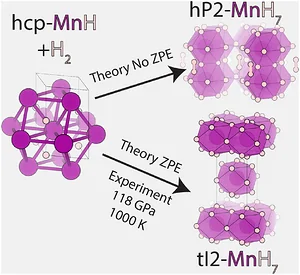

## 1 Introduction

The chemical reactivity of molecular $H_{2}$ increases dramatically under high pressure. This renders pressure a powerful tool for the synthesis of superhydrides, which are a class of materials with an exceptionally high hydrogen content (typically > 80 at.% H). Binary superhydrides emerge in many of the group I–III elements under pressure, some of which exhibit fascinating properties, such as high‐temperature superconductivity [1, 2, 3, 4, 5, 6, 7, 8, 9, 10, 11, 12, 13, 14, 15, 16, 17, 18, 19, 20, 21, 22, 23, 24, 25, 26, 27, 28]. Pressures in excess of 100 GPa are required for the synthesis of the vast majority of these materials, and it is important to produce new superhydrides at lower pressures before this class of materials can find any industrial applications.

Despite many binary hydrides forming across the group IV–XII transition metals, there has only been one reported superhydride, ${\mathrm{FeH}}_{5}$ [29]. This unique compound crystallises in a tetragonal body‐centred iron lattice (Pearson symbol tI4, space group I4/mmm), in which hydrogen forms an extended atomic network consisting of alternating ‘${\mathrm{FeH}}_{3}$’ and ‘${\mathrm{FeH}}_{7}$’ layers [29]. While high pressure generally promotes increased hydrogen uptake, ${\mathrm{FeH}}_{5}$ represents the highest hydrogen content achieved so far in a transition‐metal hydride. Transition metal hydrides are instead limited to lower stoichiometries, most commonly trihydrides such as ${\mathrm{FeH}}_{3}$, ${\mathrm{IrH}}_{3}$ and ${\mathrm{CoH}}_{3}$ [29, 30, 31], all of which contain exclusively atomic hydrogen as opposed to molecular or quasimolecular $H_{2}$.

Extended networks of atomic hydrogen are also a defining structural motif of the majority of superconducting superhydrides, and the absence of quasimolecular $H_{2}$ units is widely regarded as a key prerequisite for metallicity and superconductivity in these materials. In contrast, superhydrides of most other elements predominantly stabilise hydrogen in the form of localised quasimolecular $H_{2}$ units, and occasionally $H_{3}^{-}$ anions [1, 2, 3, 32], which generally leads to insulating behaviour. Within this framework, transition‐metal hydrides capable of sustaining dense, fully atomic hydrogen sublattices at high hydrogen content remain exceptionally rare, motivating the exploration of Mn–H compounds as potential candidates for higher‐order polyhydrides with metallic electronic structures.

In recent years, extensive quantum chemistry calculations employing density‐functional theory (DFT) have been performed for the majority of the binary metal‐hydrogen systems under high pressure [33, 34, 35]. Manganese superhydride ${\mathrm{MnH}}_{7}$ was calculated to have the lowest formation pressure of 40 GPa among all theoretically predicted transition‐metal superhydrides [36]. The hexagonal crystal structure of predicted ${\mathrm{MnH}}_{7}$ consists of the AA‐stacked graphite‐like layers of manganese atoms (Pearson symbol hP2, space group P6/mmm) with quasimolecular $H_{2}$ between these layers [35, 36]. In addition, other manganese polyhydrides, ${\mathrm{MnH}}_{2}$ (tI4, I4/mmm), ${\mathrm{MnH}}_{3}$ (tI8, I4/m) [36], and ${\mathrm{Mn}}_{3}H_{11}$ (tP3, P4/mmm) [35] have been predicted to form at similar pressures.

Previously, the manganese–hydrogen system has been studied at high pressures up to 8 GPa [37, 38]. Apart from solid solutions, together with non‐stoichiometric high‐temperature phases, the only manganese hydride known so far is ε‐${\mathrm{MnH}}_{∼1}$ with a hexagonal close‐packed (hcp) lattice [38, 39, 40, 41]. For simplicity, this phase is called hcp‐MnH henceforth. This phase possesses an antiferromagnetic order below $T_{N}$ = 350 K [42].

Here, we study the manganese–hydrogen (deuterium) system to pressures of 182 GPa. We discover three new manganese hydrides (deuterides) – ${\mathrm{MnH}}_{∼2}$, MnH(D)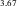, and MnH(D)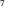. Surprisingly, the crystal structure of MnH(D)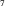 is tetragonal body‐centred (tI2, I4/mmm), rather than the previously predicted hexagonal phase (P6/mmm) with quasimolecular $H_{2}$ [35, 36]. Using our own DFT calculations, we propose a tentative arrangement of hydrogen atoms in ${\mathrm{MnH}}_{7}$, which is analogous to the ‘${\mathrm{FeH}}_{7}$’ layers in ${\mathrm{FeH}}_{5}$ [29]. Our DFT calculations demonstrate that the inclusion of the zero‐point energy (ZPE) contribution to the formation enthalpy destabilises the hypothetical P6/mmm‐${\mathrm{MnH}}_{7}$ with respect to tetragonal tI2‐${\mathrm{MnH}}_{7}$. Similarly, when ZPE is taken into account, the experimentally found cubic cP3‐${\mathrm{MnH}}_{3.67}$ replaces the predicted tetragonal tI8‐${\mathrm{MnH}}_{3}$ on the convex hull. We have investigated whether the isotope effect would stabilise different structures in manganese superdeuterides; however, even the reduced ZPE for ${\mathrm{MnD}}_{7}$ was still high enough to destabilise the hypothetical P6/mmm phase. Electrical resistance measurements revealed that cP3‐${\mathrm{MnD}}_{3.67}$ and tI2‐${\mathrm{MnD}}_{7}$ are not superconducting down to 8.5 K at 147 GPa.

## 2 Results and Discussion

### 2.1 Synthesis and Structure of ${\mathrm{MnH}}_{7}$

Upon exposing manganese to a hydrogen (deuterium) atmosphere at a pressure of 4 GPa (the lowest pressure in this study), hcp‐MnH(D) forms on the timescale of a few days at room temperature. Upon compression to 138 GPa hcp‐MnH(D) persists with no further reaction or phase transition (a typical x‐ray diffraction pattern of hcp‐MnH is shown in Figure 1a). Laser heating at pressures below 101 GPa does not induce any changes in the sample. When hcp‐MnH is heated in an excess of $H_{2}$ at pressures above 118 GPa, the sample expands dramatically (see sample photos in Table S1), indicating the formation of a new hydrogen‐rich phase. The effect of the laser heating on the crystal structure of the sample is illustrated by the changes in its x‐ray diffraction pattern shown in Figure 1b. This new hydride has a body‐centred tetragonal lattice of manganese atoms with Pearson symbol tI2 and space group I4/mmm, and an axial ratio of c/a∼2.8 (the relevant crystal structures are summarised in Table 1).

**TABLE 1:** Crystal structures of the observed manganese hydrides and deuterides with the lattice parameters interpolated to the selected pressures, together with the results of the DFT predictions.

Compound	Pressure (GPa)	Space group and lattice parameters	Atomic coordinates	Source
hcp‐MnH(D)	100	$P6_{3}/mmc$ a=2.459 Å c=3.919 Å	Mn 2c 1/3 2/3 1/4 H 2a 0 0 0	Exp
$tI4-{\mathrm{MnH}}_{∼2}$	107	I4/mmm a=2.492 Å c=8.375 Å	Mn 4e 0 0 0.152	Exp
	150	I4/mmm a=2.378 Å c=7.869 Å	Mn 4e 0 0 0.152 H1 4c 0 1/2 0 H2 4e 0 0 0.352	DFT, [36]
cP3‐MnH(D) 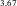	100	$Pm\bar{3}m$ a=3.570 Å	Mn 3c 0 1/2 1/2	Exp
		P4/mmm a=3.596 Å c=3.534 Å	Mn1 1a 0 0 0 Mn2 2e 0 1/2 1/2 H1 1b 0 0 1/2 H2 2f 0 1/2 0 H3 8r 0.295 0.295 0.769	DFT
tI2‐MnH(D) 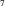	100	I4/mmm a=2.537 Å c=7.142 Å	Mn 2a 0 0 0	Exp
		Pbcn a=3.516 Å b=3.613 Å c=7.078 Å	Mn 4c 0 0.748 1/4 H1 4c 0 0.319 1/4 H2 8d 0.762 ‐0.039 0.608 H3 8d 0.282 0.459 0.616 H4 8d 0.044 0.732 0.462	DFT

**FIGURE 1:**
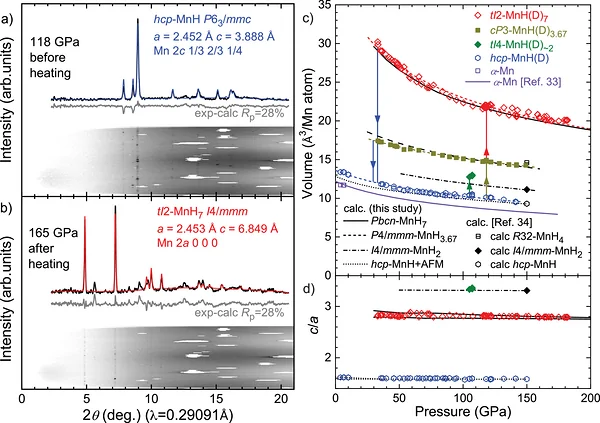
X‐ray powder diffraction patterns of a Mn–H sample at 118 GPa (a) and after laser heating at 165 GPa (b), together with the results of the Rietveld refinement of its crystal structure. The bottom panels show the raw detector images in the 2θ‐azimuth representation, the black curves are the azimuthally integrated data, the blue and red curves show the calculated contributions from the hcp‐MnH and tI2‐${\mathrm{MnH}}_{7}$ phases, respectively, and the grey curves are the refinement residuals. The peaks at 5.6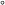, 9.1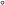, and 12.5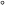 in (b) are due to unidentified impurity, MgO, and instrumental background, respectively. Volumes (c) and the axial c/a ratios (d) of the studied manganese hydrides (deuterides) as a function of pressure. The literature data for α‐Mn is taken from Ref. [43]. The results of the DFT predictions from Ref. [36] at 150 GPa are shown by the black symbols. The black curves show the results of our DFT calculations, and the curves of the other colours show the equation of state fits with the parameters indicated in Table 2. The axial ratios for Pbcn‐${\mathrm{MnH}}_{7}$, calculated with DFT, are multiplied by $\sqrt{2}$ to enable a comparison with experiment. The vertical arrows indicate the minimal pressures, at which phase transitions were observed upon compression, and the maximal pressures at which they were observed upon decompression.

The formation of this new hydride is accompanied by an expansion of the metal lattice by $\Delta V=V\left(tI2-{\mathrm{MnH}}_{x}\right)-V\left(hcp-\mathrm{MnH}\right)=12.04$ Å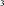/Mn atom at 118 GPa. Because the overall volume effect for the reaction should be negative according to Le Chatelier's principle, the H/Mn composition of this new hydride is larger than 6.3, based on the volume of the hydrogen gas at this pressure (4.52 Å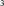/$H_{2}$) [44]. As such, the composition of this new hydride is close to H/Mn = 7.

${\mathrm{MnH}}_{7}$ persists upon subsequent compression and laser heating up to 182 GPa, which was the highest pressure reached in this study. Upon decompression, it remains stable down to 33 GPa and then decomposes into a poorly crystalline product which is presumably MnH. The V(P) dependence of tI2‐${\mathrm{MnH}}_{7}$ is shown by the open red diamonds in Figure 1c. This V(P) dependence can be fit well with the Birch–Murnaghan equation of state [45] with the parameters listed in Table 2, as shown by the dashed red curve in Figure 1c, which indicates that the compound remains fully stoichiometric and composition is pressure‐independent.

**TABLE 2:** Equation of state parameters for Mn and Mn–H phases.

Phase	Pressure (GPa)	$V_{0}$ (Å 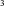 /Mn atom)	$B_{0}$ (GPa)	$B_{0}^{\prime }$	EoS type	Source
α‐Mn	0–190	12.21	158(3)	4.6(1)	Vinet	[43]
hcp‐MnH(D)	0–141	13.61(3)	201(6)	4 (fixed)	B–M	this study
cP3‐MnH(D) 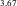	33–149	19.17(6)	267(10)	4 (fixed)	B–M	this study
tI2‐MnH(D) 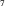	33–182	39.5(20)	62(20)	4 (fixed)	B–M	this study

### 2.2 The Role of Zero Point Energy in the Stability of ${\mathrm{MnH}}_{7}$

Given that the arrangement of the metal atoms in ${\mathrm{MnH}}_{7}$ and the '${\mathrm{FeH}}_{7}$' layers in the previously observed ${\mathrm{FeH}}_{5}$ [29] is similar, the arrangement of hydrogen atoms should also be similar. In order to elucidate the full crystal structure of ${\mathrm{MnH}}_{7}$, we assumed the arrangement of hydrogen atoms from the ‘${\mathrm{FeH}}_{7}$’ layers and optimised the resulting crystal structure with DFT at 100 GPa. The resulting Pbcn structural model is shown in Figure 2e, and the corresponding crystal structure parameters are listed in Table 1. This structure is based on the $\sqrt{2}*\sqrt{2}*1$ supercell of the experimentally observed body‐centred tetragonal unit cell of ${\mathrm{MnH}}_{7}$ with a small orthorhombic distortion (b/a=1.027), which makes this structure dynamically stable (Figure S2). The V(P) dependency and the axial ratios for Pbcn‐${\mathrm{MnH}}_{7}$, shown by the solid black curves in Figure 1c,d are in a reasonable agreement with the experimental data for tI2‐${\mathrm{MnH}}_{7}$ (open red diamonds).

**FIGURE 2:**
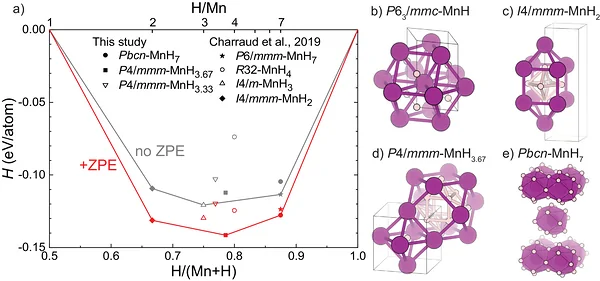
(a) Formation enthalpies of manganese hydrides at P = 100 GPa and T = 0 K, calculated with DFT with (red) and without (grey) zero‐point energy (ZPE) correction. The predicted crystal structures for I4/mmm‐${\mathrm{MnH}}_{2}$, I4/m‐${\mathrm{MnH}}_{3}$, R32‐${\mathrm{MnH}}_{4}$, and P6/mmm‐${\mathrm{MnH}}_{7}$ are taken from Ref. [36]; the rest of the structures were found in this study. (b–e) Crystal structures of the observed manganese hydrides. Manganese and hydrogen atoms are shown by the purple and pink spheres, respectively. The atomic coordinates for Mn and H atoms, also listed in Table 1, are from the XRD experiments and DFT predictions, respectively.

The formation enthalpies of Pbcn‐${\mathrm{MnH}}_{7}$ and the previously predicted P6/mmm‐${\mathrm{MnH}}_{7}$, calculated with DFT at 100 GPa, are shown by the closed circles and stars, respectively, in Figure 2a. Without ZPE, P6/mmm‐${\mathrm{MnH}}_{7}$ is more stable by 9 meV/atom as shown by the grey star.

The hydrogen motif in Pbcn‐${\mathrm{MnH}}_{7}$ is purely atomic in contrast to P6/mmm‐${\mathrm{MnH}}_{7}$, where the hydrogen motif contains quasimolecular $H_{2}$ units. This is manifested by the calculated phonon density of states of Pbcn‐${\mathrm{MnH}}_{7}$ (see Figure S3), which, in contrast to that of the hypothetical P6/mmm‐${\mathrm{MnH}}_{7}$, does not have a high‐frequency peak of quasimolecular $H_{2}$ vibrations around 550 meV. As a result, the average phonon energy, ⟨E⟩, in Pbcn‐${\mathrm{MnH}}_{7}$ is smaller than that in P6/mmm‐${\mathrm{MnH}}_{7}$ by about 8 meV. The ZPE contribution per atom is directly related to the average phonon energy, ZPE = $\frac{3}{2}\left\langle E\right\rangle$, so that ZPE(P6/mmm‐${\mathrm{MnH}}_{7}$) − ZPE(Pbcn‐${\mathrm{MnH}}_{7}$) ≈ 13 meV/atom. When ZPE contribution is added, Pbcn‐${\mathrm{MnH}}_{7}$ (red circle) replaces P6/mmm‐${\mathrm{MnH}}_{7}$ (red star) on the convex hull (see Figure 2a).

Hydrogen substitution with its heavier deuterium isotope has a dramatic effect on the vibrational and superconducting properties of hydrides. Moreover, there are thermodynamic implications of the isotope effect on ZPE, and there are at least two metals—vanadium [46] and lanthanum [15]—whose deuterides are structurally different from hydrides. To date, the studies of superdeuterides are very scarce, and only yttrium, lanthanum and cerium deuterides have been studied so far [10, 15, 18]. To explore if the predicted P6/mmm‐${\mathrm{MnH}}_{7}$ could be stabilised if its ZPE is reduced, we performed an experiment with deuterium instead of hydrogen. However, after laser heating, we observed the formation of a tI2‐${\mathrm{MnD}}_{7}$ phase, identical to tI2‐${\mathrm{MnH}}_{7}$, as illustrated by the XRD pattern in Figure S1.

### 2.3 Formation of ${\mathrm{MnH}}_{3.67}$ and ${\mathrm{MnH}}_{∼2}$

In addition to tI2‐MnH(D)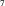, we observed a cubic phase with a Pearson symbol cP3 and a space group $Pm\bar{3}m$. The x‐ray diffraction pattern and the results of the Rietveld refinement of the crystal structure of this phase are shown in Figure S1. The crystal structure of the metal lattice of this phase is isomorphic to that of cP3‐${\mathrm{Ru}}_{3}H_{8}$ [47], and the metastable R32‐${\mathrm{MnH}}_{4}$ predicted in Ref. [36]. Additionally, a P4/mmm‐${\mathrm{Mn}}_{3}H_{11}$ compound has recently been predicted in Ref. [35], but no structural information was given. The experimental volume, shown by the yellow squares and the dashed yellow curve in Figure 1c, is somewhat smaller than the predicted volume of R32‐${\mathrm{MnH}}_{4}$ (black barred square in Figure 1c), indicating that 3 < H/Mn < 4 for this hydride. Upon decompression, this phase persists down to about 30 GPa.

The cP3 lattice features three types of interstitial sites, which can be occupied by hydrogen—1b octahedral, 3d planar square, and 8g cuboctahedral. In the predicted R32‐${\mathrm{MnH}}_{4}$ compound from Ref. [36], all of these are fully occupied. In contrast, only 8g interstitials are occupied in cP3‐${\mathrm{Ru}}_{3}H_{8}$ [47]. Because H/Mn in the cP3 manganese hydride is between that of cP3‐${\mathrm{Ru}}_{3}H_{8}$ and R32‐${\mathrm{MnH}}_{4}$, it is natural to assume that its 8g interstitials are also fully occupied, and it is either 1b or 3d interstitials which accommodate vacancies.

Our DFT calculations demonstrated that the resulting structure with the 1b vacancy was not energetically competitive, however. On the other hand, emptying all 3d interstitials would result in H/Mn = 3, which is too low from experimental volumetric considerations, drawing us to the conclusion that these interstitials are occupied partially. In order to model partial occupancy, it is necessary to break the $Pm\bar{3}m$ symmetry. The easiest way to do this is realised in the tetragonal P4/mmm subgroup, which splits the hydrogen population in 3d sites into independent 1b and 2f sites. Emptying these produced the candidate structures P4/mmm‐${\mathrm{MnH}}_{3.67}$, shown in Figure 2d, and P4/mmm‐${\mathrm{MnH}}_{3.33}$, respectively. The structural parameters for the former P4/mmm‐${\mathrm{MnH}}_{3.67}$ are listed in Table 1, and the corresponding V(P) dependence, shown by the black dashed curve in Figure 1c, is in a good agreement with the experimental data (yellow squares), which justifies the assignment of the composition of H/Mn = 3.67 for this phase. When no ZPE correction is taken into account, the formation enthalpies of all of these ${\mathrm{MnH}}_{4}$‐derived phases at 100 GPa are above that of a I4/m‐${\mathrm{MnH}}_{3}$ + P6/mmm‐${\mathrm{MnH}}_{7}$ mixture, which is on the convex hull, as shown by the grey symbols and lines in Figure 2a. However, when ZPE is included (red symbols and lines in Figure 2a), I4/m‐${\mathrm{MnH}}_{3}$ is replaced by P4/mmm‐${\mathrm{MnH}}_{3.67}$ on the convex hull.

In one of our samples with a hydrogen deficiency, we observed the formation of yet another phase after laser heating at 126 GPa. As evidenced from the refinement of its crystal structure shown in Figure S4a, this phase is isomorphic to ${\mathrm{FeH}}_{2}$ [48], and to the previously predicted I4/mmm‐${\mathrm{MnH}}_{2}$ [36] (Figure 2c). It should be noted, however, that the volume of this phase, shown by the solid olive diamonds in Figure 1c is somewhat higher than the volume of I4/mmm‐${\mathrm{MnH}}_{2}$ calculated with DFT in Ref. [36] (black diamond) and this study (black dash‐dotted curve). Volumetric considerations indicate that the hydrogen content of this phase is H/Mn > 2.15. We tried to explore the possible incorporation of extra hydrogen in the crystal structure of I4/mmm‐${\mathrm{MnH}}_{2}$ with DFT‐based crystal structure search but did not find any energetically feasible candidate structures. For this reason, we call this phase ${\mathrm{MnH}}_{∼2}$.

### 2.4 Electrical Conductivity of Manganese Hydrides

The electron density of states of the experimentally observed manganese hydrides, calculated with DFT, is shown in Figures 3a and S5. Our calculations indicate that hcp‐MnH, I4/mmm‐${\mathrm{MnH}}_{2}$, and P4/mmm‐${\mathrm{MnH}}_{3.67}$ are metallic, and Pbcn‐${\mathrm{MnH}}_{7}$ is a semiconductor with a bandgap 0.6 eV (PBE) or 2.5 eV (HSE06) at 100 GPa. The calculations also correctly reproduce the antiferromagnetic ground state of hcp‐MnH below 50 GPa (Figure S5a) in agreement with the literature [42].

**FIGURE 3:**
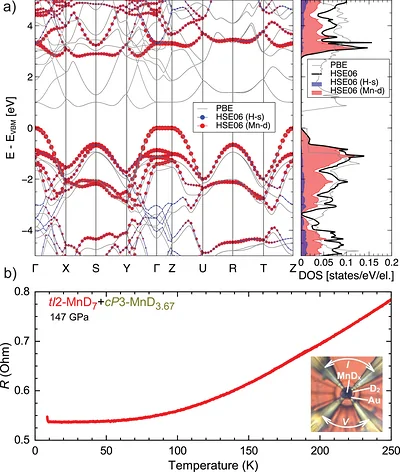
(a) Electronic dispersion curves and density of states of Pbcn‐${\mathrm{MnH}}_{7}$, calculated at 100 GPa with PBE (grey) and HSE06 functionals (blue and red show the projection onto the H‐s and Mn‐d states, respectively). (b) Temperature dependence of the electrical resistance of the ${\mathrm{MnD}}_{7}$ + ${\mathrm{MnD}}_{3.67}$ sample upon warming from 8.5 K at the pressure of 147 GPa. Photomicrograph of the samples with the electrode configurations are shown in the insets.

To explore conductivity experimentally, we have measured the electrical resistance of our ${\mathrm{MnD}}_{7}$ + ${\mathrm{MnD}}_{3.67}$ sample with a four‐probe technique at 147 GPa, as shown in Figure 3b. We observed that the sample is metallic, but no anomalies were detected above 8.5 K. Given that our calculations indicate that ${\mathrm{MnH}}_{7}$ is a semiconductor at such pressures, most likely the conductivity arises from metallic ${\mathrm{MnD}}_{3.67}$. Previously, no superconductivity has been detected in ${\mathrm{FeH}}_{5}$ above 1.5 K [49].

Electrical resistance measurements of a ${\mathrm{MnH}}_{∼2}$ + MnH at 107 GPa revealed a drop at 4.7 K (Figure S4b), which could be indicative of a superconductivity onset. On the other hand, we calculated the expected $T_{c}$ of hcp‐MnH and stoichiometric I4/mmm‐${\mathrm{MnH}}_{2}$ at 100 GPa using density functional perturbation theory (DFPT), but found the $T_{c}$ to be less than 0.1 K. Further studies would be insightful to account for this discrepancy and to explore potential superconductivity in these manganese hydrides.

## 3 Conclusions

In summary, we have explored the Mn‐H system under high pressures up to 182 GPa, discovering three new compounds, ${\mathrm{MnH}}_{7}$, ${\mathrm{MnH}}_{3.67}$ and ${\mathrm{MnH}}_{∼2}$. Surprisingly, the body‐centred tetragonal crystal structure of ${\mathrm{MnH}}_{7}$ is different from the lowest enthalpy structure found with DFT. Instead, our calculations demonstrate that the inclusion of ZPE is essential to renormalise the energy rankings and correctly predict the crystal structure of this new material. Intrinsically stabilised by nuclear quantum effects, ${\mathrm{MnH}}_{7}$ is only the second transition metal superhydride to be discovered and could indicate that more hydrogen‐rich transition metal compounds could be synthesised at high pressure high‐temperature conditions.

## Conflicts of Interest

The authors declare no conflicts of interest.

## Supporting information


**Supporting File**: anie74012‐sup‐0001‐SuppMat.pdf.

## Data Availability

The data that support the findings of this article are available in the Supporting Information, which includes Refs. [50, 51, 52, 53, 54, 55, 56, 57, 58, 59, 60, 61, 62, 63, 64], the experimental details, sample table, x‐ray diffraction data, electrical resistance measurements, and DFT calculations. Raw data will be made available upon request.
