# Supplementary material for: High‐Pressure Synthesis of Quantum‐Stabilised Manganese Superhydride MnH7
Source: Angew Chem Int Ed Engl. 2026 Aug 22;65(41):e9526719. doi: 10.1002/anie.9526719 (PMC13638122; doi:10.1002/anie.9526719)
Supplement: Supplementary file 1 [file ANIE-65-e9526719-s001.pdf]

Supplementary Materials for

High-Pressure Synthesis of Quantum-Stabilised Manganese Superhydride MnH_7

Mikhail A. Kuzovnikov, Hannah A. Shuttleworth, Israel Osmond, Huixin Hu, Elvita Meskauskaite, Bole Chen, Callum Stevens, Andrew Huxley, Andreas Hermann, Miriam Peña-Alvarez, and Ross T. Howie

Experimental Details

Sample Preparation

The performed experiments are summarized in Table S1. We used the symmetric Mao-type, and our own custom designed "SH" type diamond anvil cells equipped with (100)-oriented diamonds of the Boehler-Almax design. The diamonds with the culet diameters of 50-70 μm were beveled at 8° to a diameter of 300-350 μm. We used both rhenium gaskets and insulating gaskets made of an MgO + epoxy composite. The gaskets were indented to a thickness of 6-8 μm, and a hole with a diameter of 80% of the culet diameter was drilled with a custom-built UV laser drilling setup. A grain of gold powder was loaded into the sample chamber, and the pressure in x-ray diffraction experiments was determined from the equation of state of gold from Ref. [dorfman2012]. In run 1, we used the MgO equation of state for the pressure determination [dorfman2012]. Freshly powdered manganese sample (USSR, 99.99%) was loaded, and subsequently H_2 (BOC, 99.9995%) or D_2 (BOC, 99.8%) gas was loaded at a pressure of 0.2 GPa. The gases were always in excess unless otherwise stated, and they served both as a reagent and as a pressure-transmitting medium. After reaching the desired pressures, the samples were heated with a continuous-wave 1070 nm Yb-doped fiber laser (YLR-200-AC, IPG Photonics) in a custom-built laser heating system, or at the respective synchrotron beamlines. The temperature in the laser spot can be estimated to be of the order of 1000 K.

Electrical Measurements

For the resistance measurements, four electrodes were sputtered onto the diamond surface with a Korvus HEX magnetron sputtering system through a custom-made mask in van der Pauw geometry. A layer of about 200 nm of tungsten was deposited first to ensure good adhesion, and then a 100 nm gold layer was deposited to decrease the resistance and to protect tungsten from reacting with hydrogen. Before sample loading, the diamond culets were chemically etched to remove metals deposited between the electrodes until the resistance between the electrodes became infinite. During the experiments, the resistances between any of the electrodes were between 30-1000 Ω. Electrical resistance was measured in four-probe configuration with the amplitudes of the excitation current of 141.42 (run 3) or 100 μA (run 4) supplied by a Keithley 6221 AC source at the frequency of 83 (run 3) or 19 Hz (run

4). Voltage was detected using a Stanford SR830 Lock-In amplifier. A Cernox thermometer attached to the DAC body was used to measure the temperature.

X-ray Diffraction Measurements

Angular-dispersive powder X-ray diffraction experiments were performed at the Extreme Conditions Beamline (ECB, P02.2) at PETRA-III, Hamburg, Germany [liermann2015], at the ID15-b [garbarino2024] and ID27 [mezouar2024] beamlines at the ESRF, Grenoble, France, and at the BL10XU beamline at SPring-8, Huogo, Japan [hirao2020]. We collected 2D maps of each sample with the typical size of 5x5 to 9x9 after each laser heating cycle in order to select the best frames for subsequent structural refinement. Typically, in each collected frame the cell was oscillated around vertical axis by ±10-15°, and the 2D diffraction image was collected for 10 s.

At P02.2, we used an incident X-ray beam with $E \approx 42.5$ keV ($\lambda \approx 0.292$ Å), focused to ~ 2 μm spot using Kirkpatrick-Baez mirrors. Diffraction images were recorded using Perkin Elmer XRD1621 detector with a sample-to-detector distance (SDD) of ~ 400 mm, calibrated with a CeO_2 standard. At ID15-b an incident beam with $E \approx 30.3$ keV ($\lambda \approx 0.4097$ Å) was focused to ~1 μm spot. The diffraction patterns were collected with EIGER2 X 9M detector with SDD ≈ 181 mm, calibrated using a Si powder standard. At ID27 an incident beam with $E \approx 33.2$ keV ($\lambda \approx 0.3738$ Å) was focused to a ~ 0.5 μm spot, and EIGER2 X 9M CdTe detector was used for data collection. The SDD ≈ 190 mm was calibrated with a CeO_2 standard. At BL10XU an incident beam with $E \approx 29.8$ keV ($\lambda \approx 0.4161$ Å) was collimated down to ~ 3 μm. We used Perkin Elmer XRD1621 detector with SDD ≈ 300 mm, calibrated with a CeO_2 standard.

The SDD, detector orientation and wavelength calibration, primary processing, azimuthal integration and background subtraction were done with the DIOPTAS v0.5.5 software [prescher2015]. Phase analysis and Rietveld refinements were performed using the POWDERCELL 2.4 program [kraus1996].

Computational Methods

Density functional theory (DFT) calculations were performed for structure optimizations and phonon calculations, using the Vienna Ab initio simulation package (VASP [kresse1996]) together with the "hard" hydrogen and the 13-electron manganese PAW pseudopotentials [kresse1999]. Electronic exchange-correlation

effects were described with the Perdew-Burke-Ernzerhof (PBE) functional [perdew1996]. Geometry optimisations used a plane wave cutoff energy of E_c = 800 eV, and regular Brillouin zone sampling grids with linear density 40 1/Å^{-1} [monkhorst1976]. Geometries were optimised until residual forces were below 1 meV/Å, and residual stresses below 0.1 GPa. Additionally, the electronic properties were calculated using the Heyd-Scuseria-Ernzerhof (HSE06) hybrid functional [krukau2006].

Crystal structures for manganese polyhydrides were taken from a previous structure search [charraud2019]. Their formation enthalpies were evaluated against manganese monohydride, ε-MnH, in a hexagonal close packed (*hcp*) structure; and H_2 in both $P6_3/m$ (model for phase I) and $C2/c$ (model for phase III) structures.

Magnetic calculations including spin-orbit coupling could stabilise the antiferromagnetic structure for MnH reported by Irodova et al. [irodova1987], and found that magnetic moments disappeared at pressures of 50 GPa and above.

The non-stoichiometric compounds MnH_{2+x} and MnH_{4-y} were modelled by (i) adding hydrogen atoms to the tetrahedral sites of the $I4/mmm$-MnH_2 structure (four models, x = 0.25, 0.50, 0.75, 1.00); and (ii) removing hydrogen atoms from the Pm-$3m$-MnH_4 structure (three models, y = 1/3, 2/3, 1).

Phonon calculations used the finite displacement method and suitable supercells to determine the real space force constant matrix, as implemented in the phonopy package [togo2023, togo2023a]. The phonon dispersion curves and density of states for *hcp*-MnH, $I4/mmm$-MnH_2, $P4/mmm$-$MnH_{3.67}$, and $Pbcn$-MnH_7, calculated at 100 GPa, are shown in Fig. S2. For harmonic-level free energies, the vibrational zero-point energies and entropies were added to DFT ground state enthalpies.

Electron-phonon coupling (EPC) and T_c of $P6_3/mmc$-MnH and $I4/mmm$-MnH_2 at 100 GPa were calculated with the QUANTUM ESPRESSO software [giannozzi2009]. A kinetic energy cutoff of 80 Ry and a charge density cutoff of 1000 Ry were used for plane wave basis. In the EPC calculation, the Brillouin zone sampling used a 6×6×6 q-mesh and a 24×24×24 K-mesh. T_c is estimated based on the Allen-Dynes modified McMillan equation: $T_c = \frac{\omega_{log}}{1.2}\exp\left(\frac{-1.04(1+\lambda)}{\lambda-\mu^*(1+0.62\lambda)}\right)$, where the Coulomb potential parameter was set as μ^* = 0.13.

Table S1. List of samples synthesized and studied in this work. The pressures indicated below were determined with the equation of state of gold and MgO [dorfman2012]. The indicated pressures of laser heating were measured after it.

No.	Photo (reflection+transmission)	Synthesis conditions	*R*(*T*)	Phase analysis results
1	At 4.6 GPa 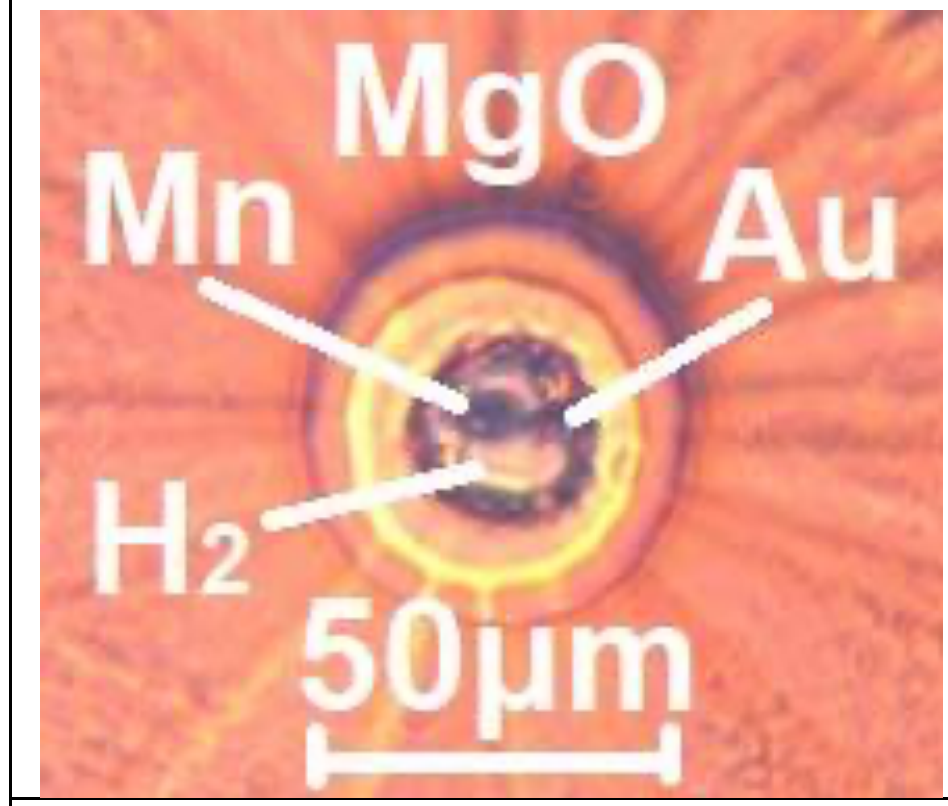	$Mn+H_2$ compressed to 75 GPa and laser heated	n/a	*hcp*-MnH with *a*=2.496Å *c*=3.972Å. XRD pattern at 117 GPa is shown in Fig. 1a.
	n/a	Compressed and laser heated at 121 GPa	n/a	*tI*2-MnH_7 with *a*=2.508Å *c*=7.038Å+*hcp*-MnH with *a*=2.456Å *c*=3.872Å
	n/a	Laser heated at 169 GPa and compressed to 182 GPa	n/a	*tI*2-MnH_7 with *a*=2.433Å *c*= 6.788Å. XRD pattern at 165 GPa is shown in Fig. 1b.
	n/a	Decompressed to 29 GPa	n/a	*tI*2-MnH_7 decomposed and the sample amorphized below 33 GPa
2	At 7.4 GPa	$Mn+H_2$ compressed to 95 GPa	n/a	*hcp*-MnH with *a*=2.454Å *c*=3.926Å

	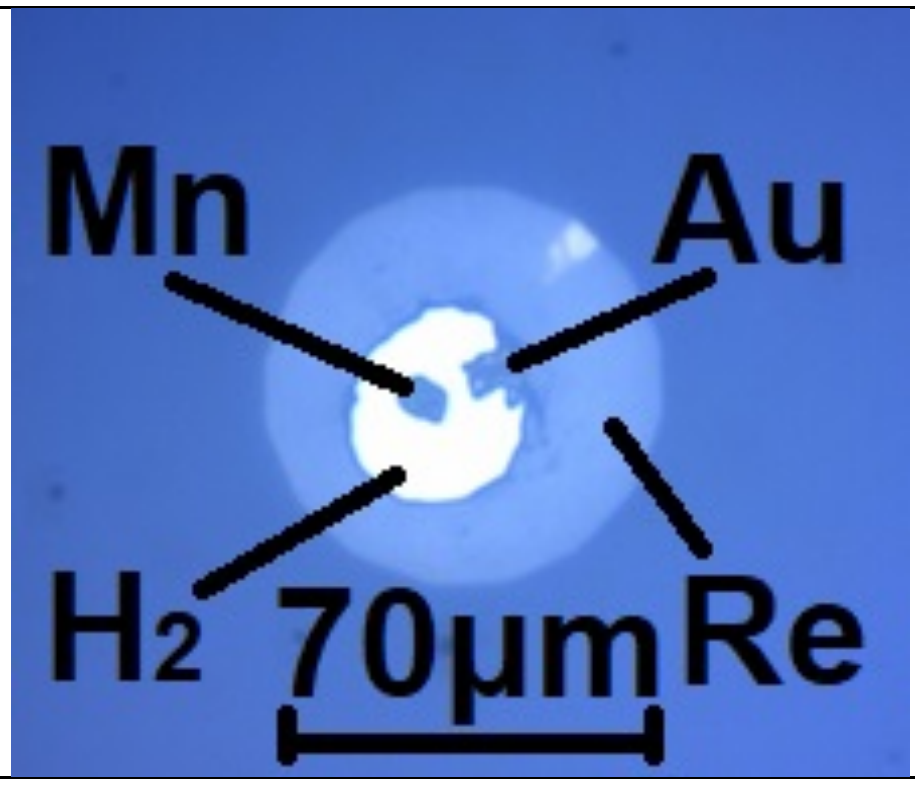			
	n/a	Laser heated at 118 GPa	n/a	*tI*2-MnH_7 with *a*=2.492Å *c*=7.007Å+*cP*3-$MnH_{3.67}$ with *a*=3.546Å
	n/a	Decompressed to 30 GPa	n/a	The sample decomposed below 33 GPa
3	before heating 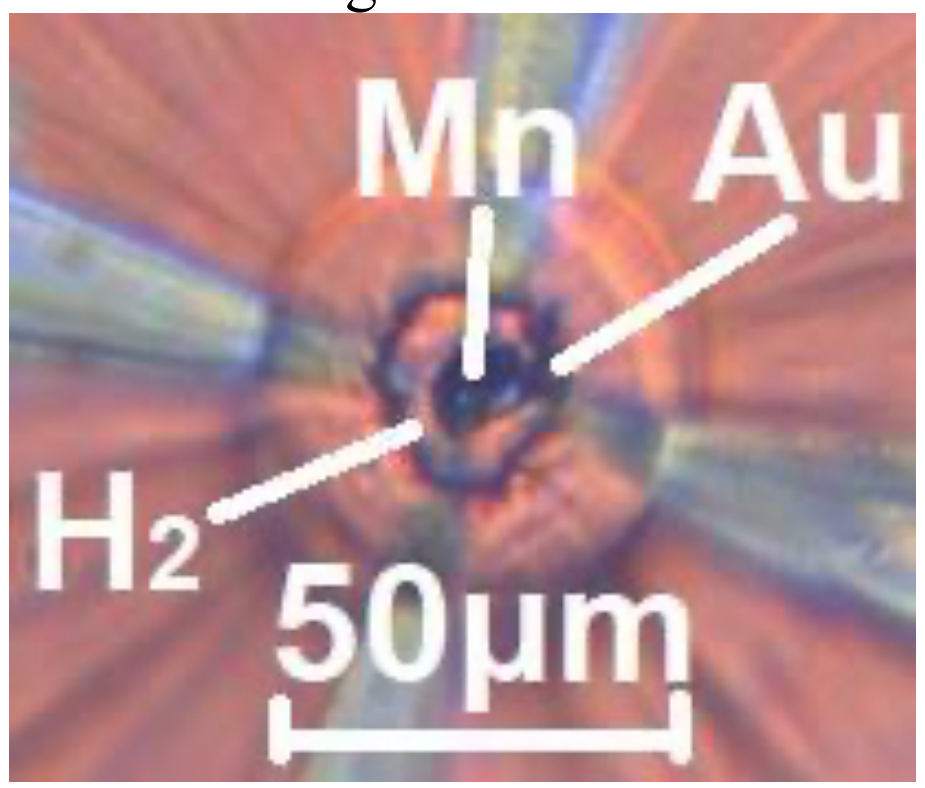	Mn+H_2 compressed to 124 GPa	n/a	n/a
	after heating	Laser heated at 126 GPa	*R*(300 K)=0.40-0.74 Ω. Resistance drop at 4.7 K at 107 GPa, see Fig.	At 107 GPa: *hcp*-MnH with *a*=2.460Å *c*=3.935Å+*tI*4-$MnH_{\sim 2}$

	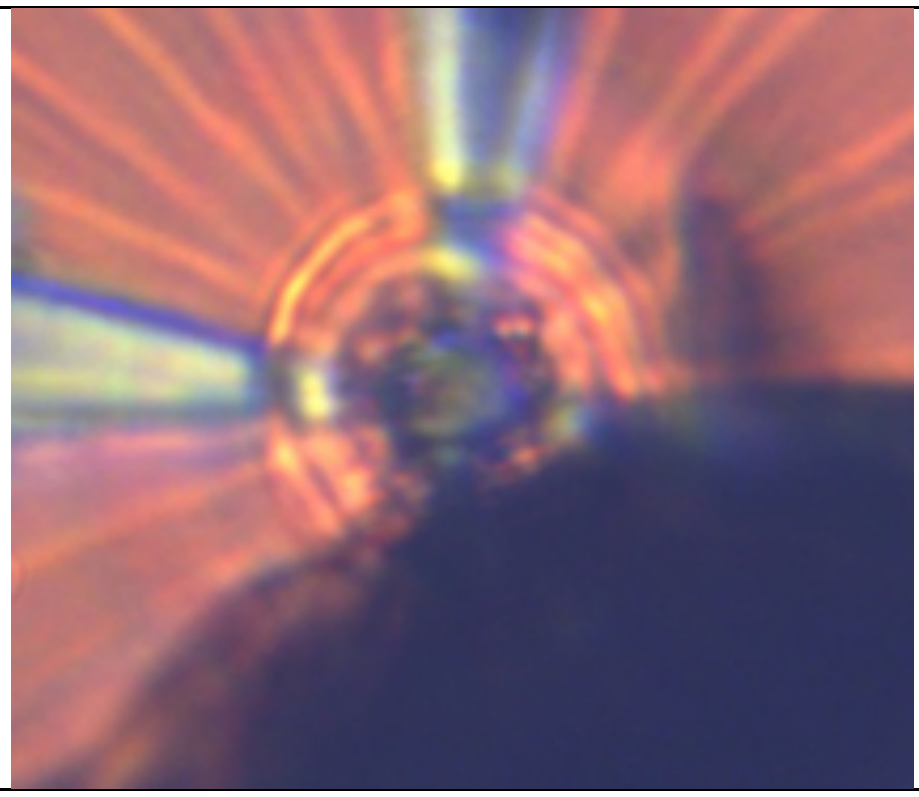		S4b	with a=2.495Å c=8.339Å, see Fig. S4a
4	after first heating<br>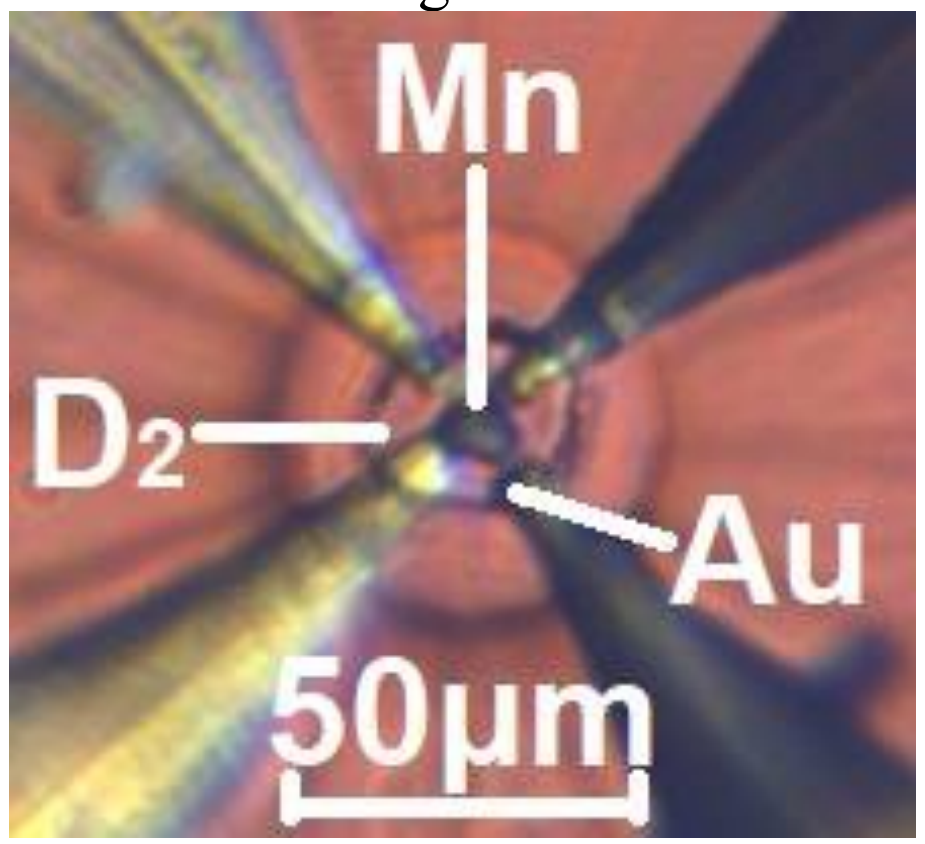 	$Mn+D_2$ compressed and laser heated at 138 GPa	n/a	*hcp*-MnD with a=2.404Å c=3.827Å+traces of *tI*2-MnD_7 with a=2.459Å c=6.899Å
	after second heating	Laser heated at 142 GPa	R(300 K)=0.84 Ω. No anomalies down to 8.5 K at 147 GPa, see Fig. 3b	*tI*2-MnD_7 with a=2.454Å c=6.873Å+*cP*3-$MnD_{3.67}$ with a=3.504Å, see Fig. S1

	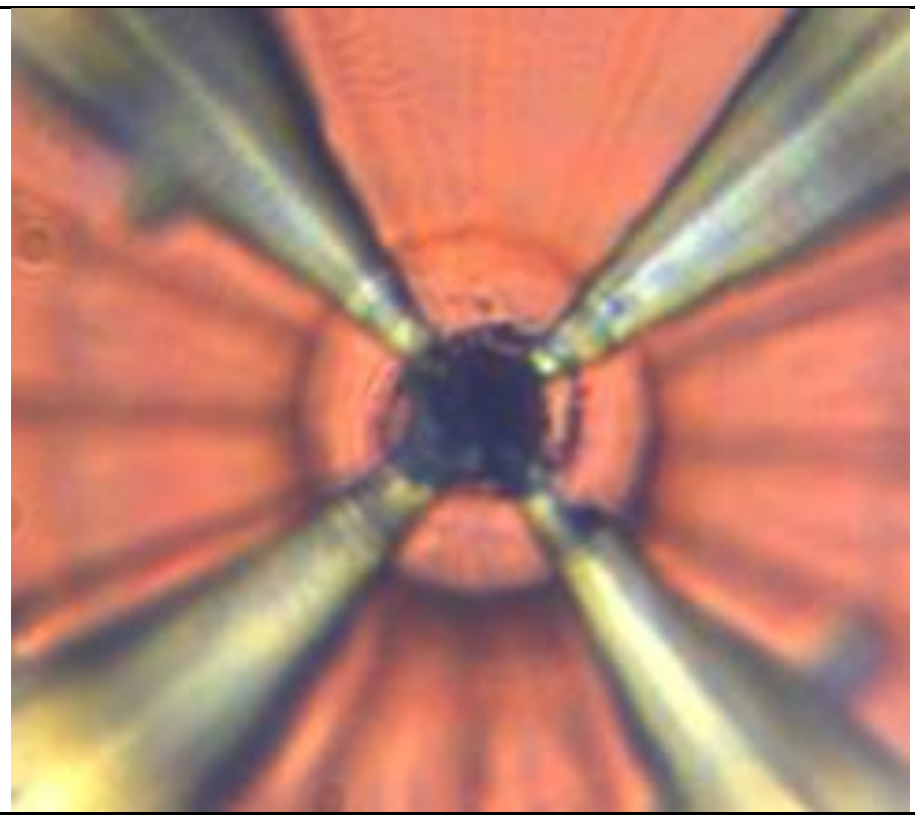			
	n/a	Decompressed to 54 GPa	n/a	*tI*2-MnD_7 with *a*=2.680Å *c*=7.508Å+*cP*3-$MnD_{3.67}$ with *a*=3.656Å

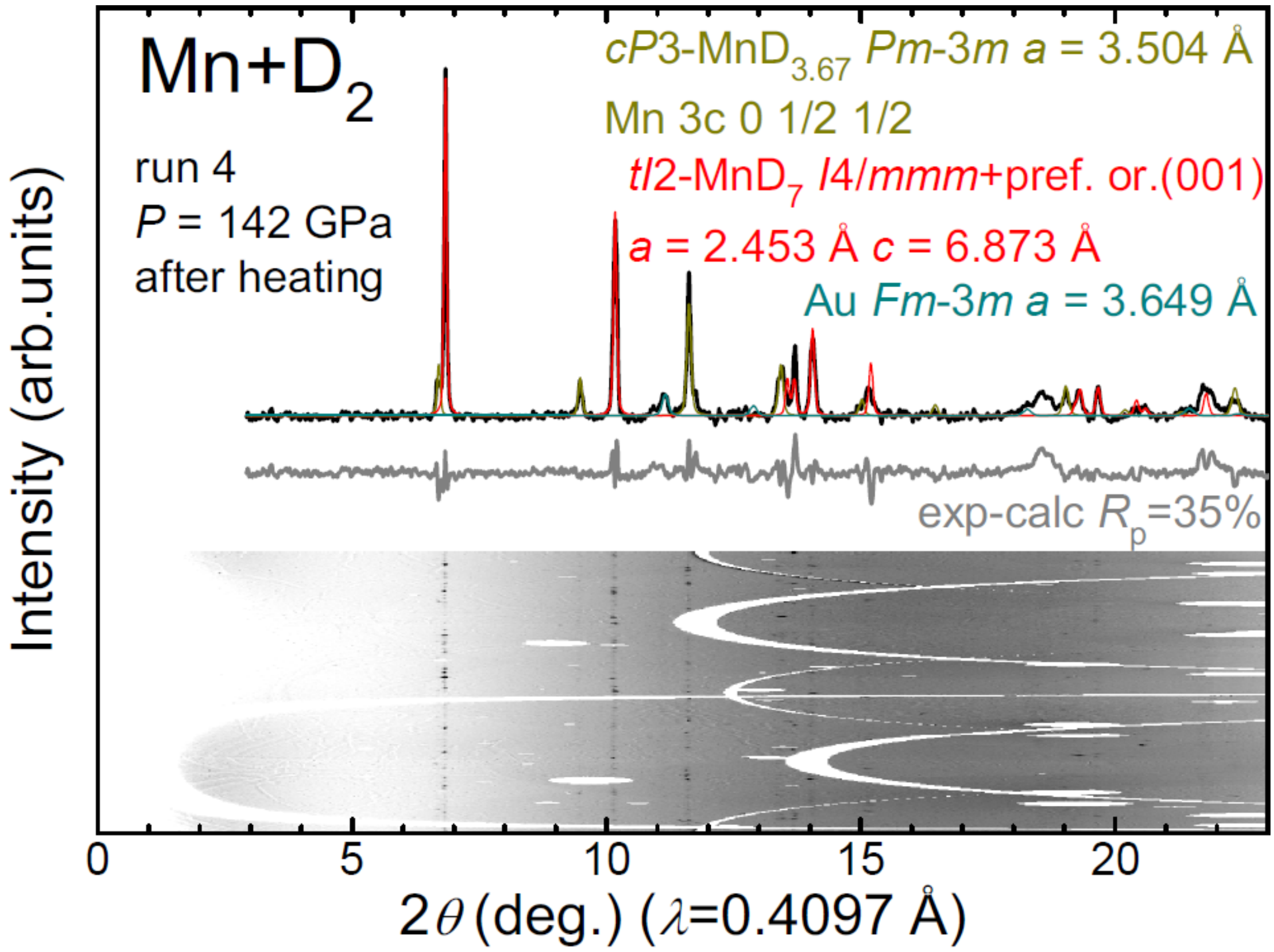

Fig. S1. X-ray powder diffraction pattern of a phase mixture *tI*2-MnD_7 + *cP*3-$MnD_{3.67}$ produced after laser heating at 142 GPa in run 4, and the results of the Rietveld refinement. The black curve is the experimental structural factor, the red and yellow curves show the calculated contributions from *tI*2-MnD_7 and *cP*3-$MnD_{3.67}$, respectively, and the grey curve is the refinement residual.

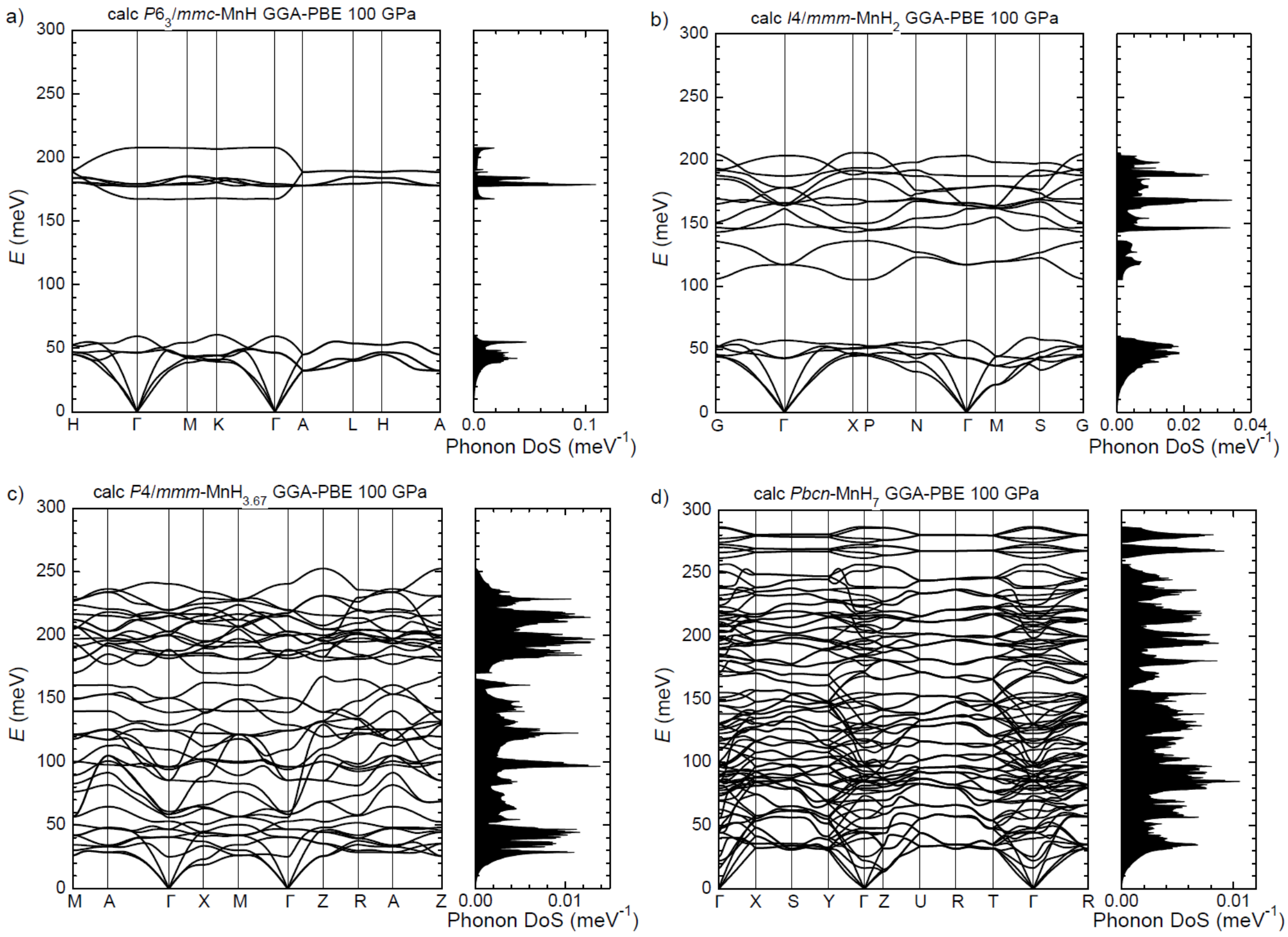

Fig. S2. Phonon dispersion curves and density of states of (a) *hcp*-MnH, (b) *I*4/*mmm*-MnH_2, (c) *P*4/*mmm*-$MnH_{3.67}$, and (d) *Pbcn*-MnH_7, calculated with DFT at 100 GPa.

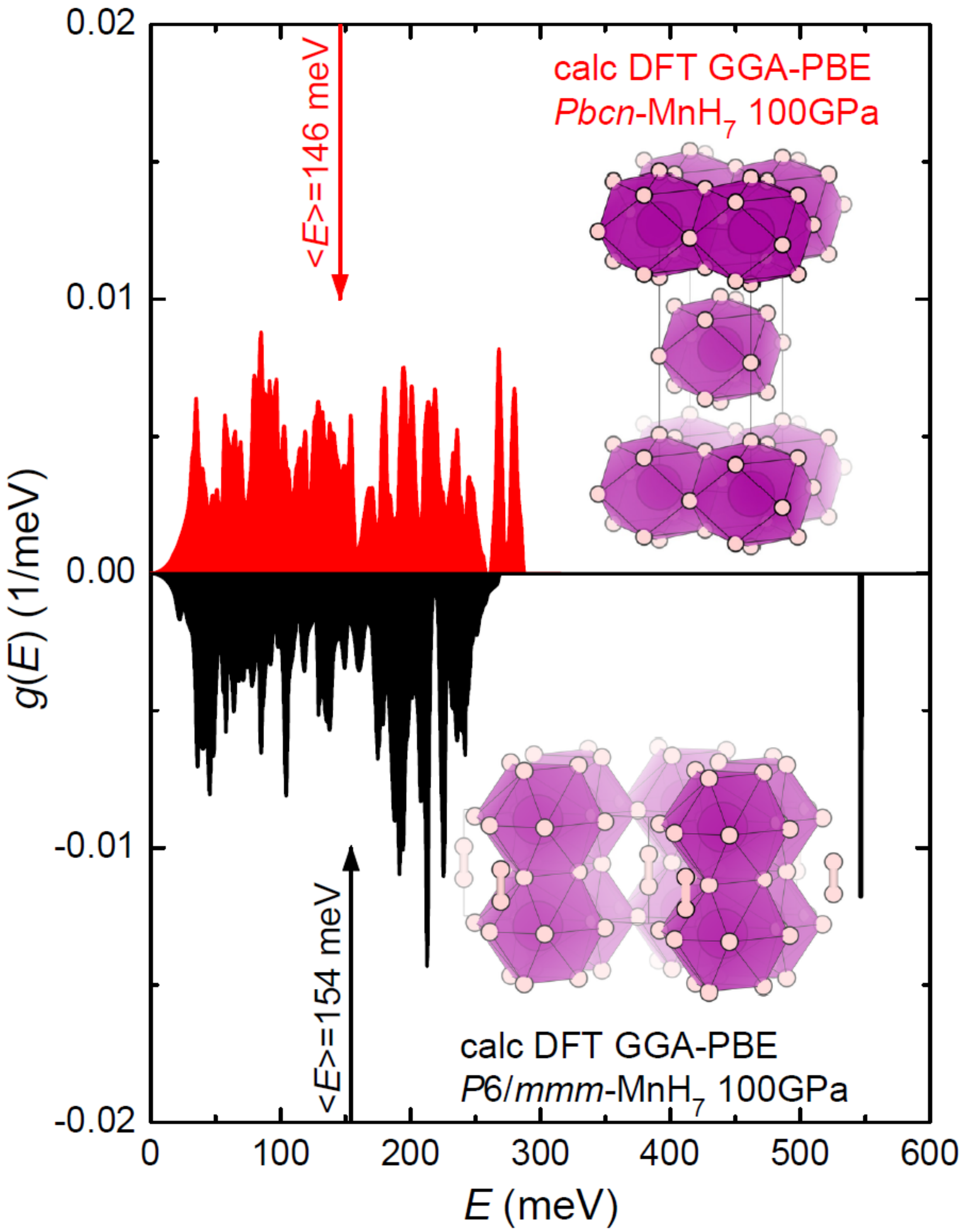

Fig. S3. Phonon density of states of the presently found *Pbcn*-MnH_7 candidate structure (top panel), and that of the hypothetical "*P6/mmm*-MnH_7" from Ref. [charraud2019] (bottom panel), calculated with DFT. In the latter, a sharp high-frequency peak at 540 meV originates from the vibrations of the quasimolecular H_2 units. The arrows show the average vibrational frequencies.

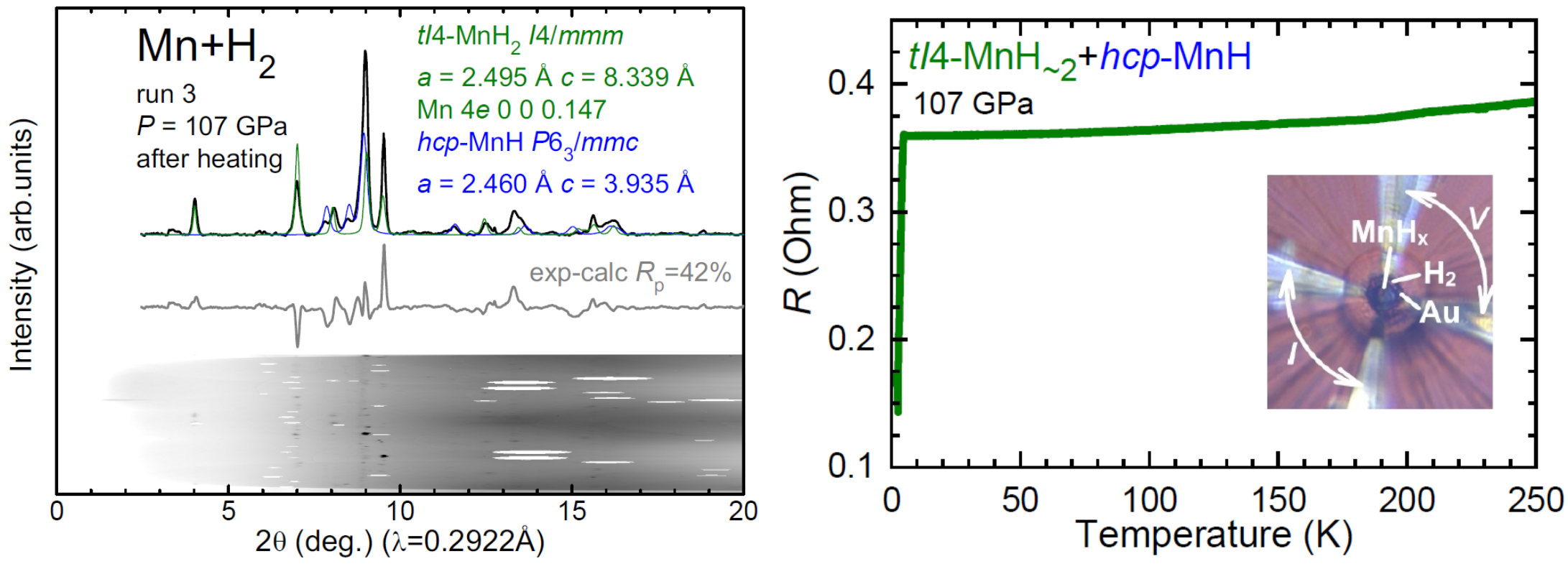

Fig. S4. (a) X-ray powder diffraction pattern of a phase mixture *tI*4-$MnH_{\sim 2}$ + *hcp*-MnH produced after laser heating at 126 GPa in run 3, and the results of the Rietveld refinement. The black curve is the experimental structural factor, the olive and blue curves show the calculated contributions from *tI*4-$MnH_{\sim 2}$ and *hcp*-MnH, respectively, and the grey curve is the refinement residual. (b) Temperature dependence of the electrical resistance of the $MnH_{\sim 2}$ + MnH sample in run 3 upon cooling at 107 GPa, showing at anomaly at 4.7 K.

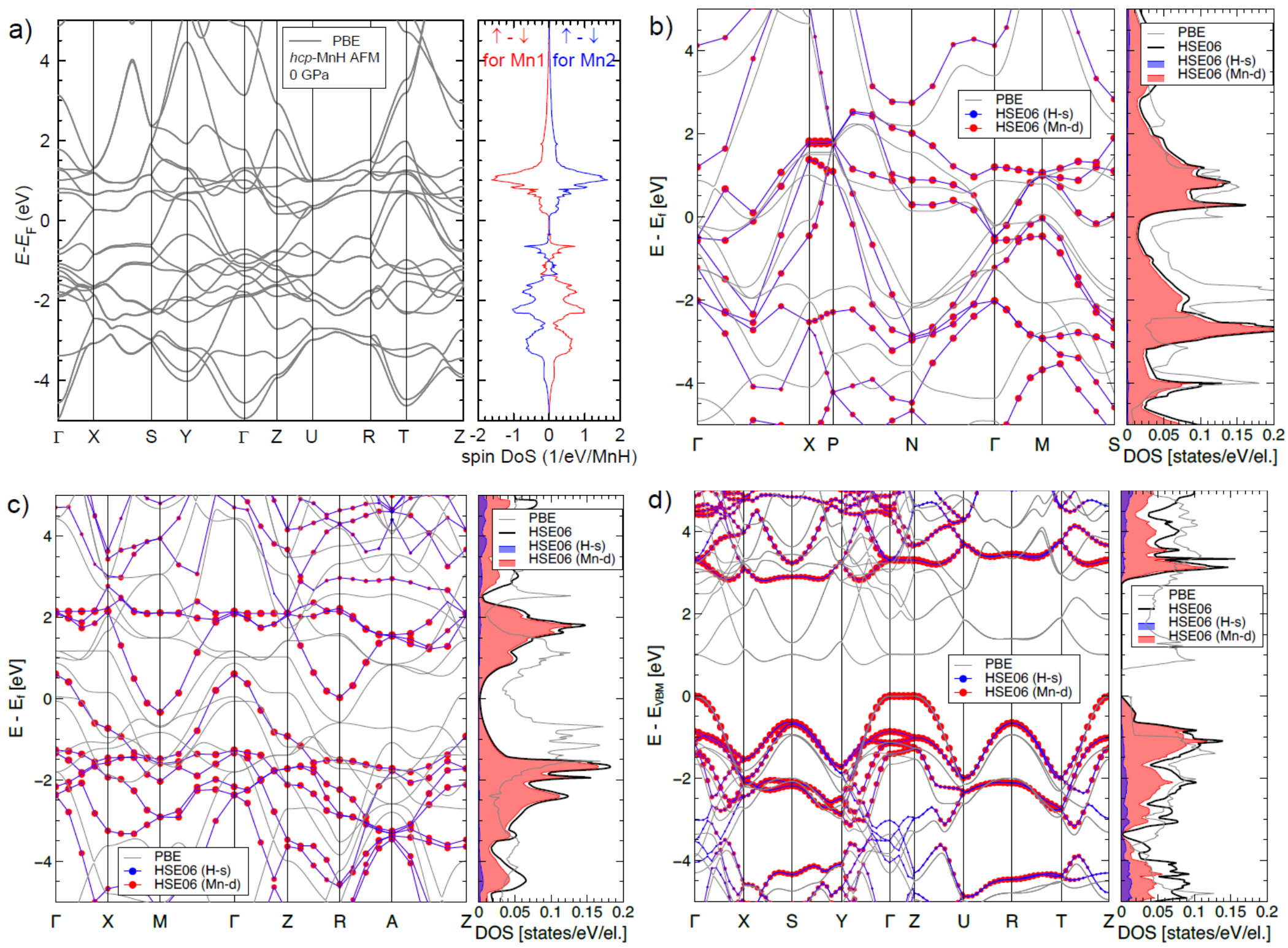

Fig. S5. Electron dispersion curves and density of states of (a) *hcp*-MnH, (b) *I*4/*mmm*-MnH_2, (c) *P*4/*mmm*-$MnH_{3.67}$, and (d) *Pbcn*-MnH_7, calculated with DFT. PBE and HSE06 functionals were used for *hcp*-MnH at 0 GPa, and for the rest of the hydrides at 100 GPa, respectively.

[dorfman2012] S. M. Dorfman, V. B. Prakapenka, Y. Meng, and T. S. Duffy, Intercomparison of pressure standards (Au, Pt, Mo, MgO, NaCl and Ne) to 2.5 Mbar, J. Geophys. Res.: Solid Earth **117**, B08210 (2012). https://doi.org/10.1029/2012JB009292

[liermann2015] H.-P. Liermann et al, The Extreme Conditions Beamline P02.2 and the Extreme Conditions Science Infrastructure at PETRA III, J. Synchrotron Radiat. **22**, 908−924 (2015). https://doi.org/10.1107/S1600577515005937

[garbarino2024] G. Garbarino, M. E. Hanfland, S. Gallego-Parra, A. D. Rosa, M. Mezouar, D. Duran, K. Martel, E. Papillon, T. Roth, P. Got, and J. Jacobs, Extreme conditions X-ray diffraction and imaging beamline ID15B on the ESRF extremely brilliant source, High Pressure Res. **44**, 199-216 (2024). https://doi.org/10.1080/08957959.2024.2379294

[mezouar2024] M. Mezouar et al, The high flux nano-X-ray diffraction, fluorescence and imaging beamline ID27 for science under extreme conditions on the ESRF Extremely Brilliant Source, High Pressure Res. **44**, 171–198 (2024). https://doi.org/10.1080/08957959.2024.2363932

[hirao2020] N. Hirao, S. I. Kawaguchi, K. Hirose, K. Shimizu, E. Ohtani, and Y. Ohishi, New developments in high-pressure X-ray diffraction beamline for diamond anvil cell at SPring-8, Matter Radiat. Extremes **5**, 018403 (2020). https://doi.org/10.1063/1.5126038

[prescher2015] C. Prescher and V. B. Prakapenka, DIOPTAS: a program for reduction of two-dimensional X-ray diffraction data and data exploration, High Pressure Res. **35**, 223-230 (2015). https://doi.org/10.1080/08957959.2015.1059835

[kraus1996] W. Kraus and G. Nolze, POWDER CELL - a program for the representation and manipulation of crystal structures and calculation of the resulting X-ray powder patterns, J. Appl. Cryst. **29**, 301–303 (1996). https://doi.org/10.1107/S0021889895014920

[kresse1996] G. Kresse and J. Furthmüller, Efficient iterative schemes for ab initio total-energy calculations using a plane-wave basis set, Phys. Rev. B **54**, 11169 (1996). https://doi.org/10.1103/PhysRevB.54.11169

[kresse1999] G. Kresse and D. Joubert, From ultrasoft pseudopotentials to the projector augmented-wave method, Phys. Rev. B **59**, 1758 (1999). https://doi.org/10.1103/PhysRevB.59.1758

[perdew1996] J. P. Perdew, K. Burke, and M. Ernzerhof. Generalized gradient approximation made simple. Phys. Rev. Lett. **77**, 3865 (1996). https://doi.org/10.1103/ PhysRevLett.77.3865

[monkhorst1976] H. J. Monkhorst and J. D. Pack, Special points for Brillouin-zone integrations, Phys. Rev. B **13**, 5188 (1976). https://doi.org/10.1103/PhysRevB.13.5188

[krukau2006] A. V. Krukau, O. A. Vydrov, A. F. Izmaylov, and G. E. Scuseria, Influence of the exchange screening parameter on the performance of screened hybrid functionals, J. Chem. Phys. **125**, 224106 (2006). https://doi.org/10.1063/1.2404663

[charraud2019] J.-B. Charraud, G. Geneste, and M. Torrent, Manganese hydrides and superhydrides at high pressure, Phys. Rev. B **100**, 224102 (2019). https://doi.org/10.1103/PhysRevB.100.224102

[irodova1987] A. V. Irodova, V. P. Glazkov, V. A. Somenkov, S. Sh. Shil'shtein, V. E. Antonov, and E. G. Ponyatovskii, Neutron diffraction study of antiferromagnetic ordering in manganese hydrides, Sov. Phys. Solid State **29**, 1562–1565 (1987)

[togo2023] A. Togo, L. Chaput, T. Tadano, and I. Tanaka, Implementation strategies in phonopy and phono3py, J. Phys.: Condens. Matter **35**, 353001 (2023). https://doi.org/10.1088/1361-648X/acd831

[togo2023a] A. Togo, First-principles Phonon Calculations with Phonopy and Phono3py, J. Phys. Soc. Jpn. **92**, 012001 (2023). https://doi.org/10.7566/JPSJ.92.012001

[giannozzi2009] P. Giannozzi, S. Baroni, N. Bonini, M. Calandra, R. Car, C. Cavazzoni, D. Ceresoli, G. L. Chiarotti, M. Cococcioni, I. Dabo et al., QUANTUM ESPRESSO: A modular and open-source software project for quantum simulations of materials, J. Phys.: Condens. Matter **21**, 395502 (2009). https://doi.org/10.1088/0953-8984/21/39/395502
